# Overcoming Challenges in Silicon Anodes: The Role of Electrolyte Additives and Solid-State Electrolytes

**DOI:** 10.3390/mi16070800

**Published:** 2025-07-09

**Authors:** Jinsik Nam, Hanbyeol Lee, Oh B. Chae

**Affiliations:** School of Chemical, Biological and Battery Engineering, Gachon University, Seongnam-si 13120, Republic of Korea; hhyyttgg2@gachon.ac.kr (J.N.); byulbbang99@gachon.ac.kr (H.L.)

**Keywords:** silicon, electrolytes, additives, solid-state electrolytes, lithium-ion batteries

## Abstract

Silicon-based anodes have emerged as promising candidates for advanced lithium-ion batteries (LIBs) owing to their outstanding lithium storage capacity; however, the commercial implementation of silicon-based anodes is hindered primarily by their significant volumetric changes and the resulting solid electrolyte interphase (SEI) instability during the lithiation/delithiation process. To overcome these issues, electrolyte optimization, particularly through the use of functional additives and solid-state electrolytes, has attracted significant research attention. In this paper, we review the recent developments in electrolyte additives, such as vinylene carbonate, fluoroethylene carbonate, and silane-based additives, and new additives, such as dimethylacetamide, that improve the SEI stability and overall electrochemical performance of silicon-based anodes. We also discuss the role of solid electrolytes, including oxides, sulfides, and polymer-based systems, in mitigating the volume changes in Si and improving safety. Such approaches can effectively enhance both the longevity and capacity retention of silicon-based anodes. Despite significant progress, further studies are essential to optimize electrolyte formulation and solve interfacial problems. Integrating these advances with improved electrode designs and anode materials is critical for realizing the full potential of silicon-based anodes in high-performance LIBs, particularly in electric vehicles and portable electronics.

## 1. Introduction

Despite significant advancements in lithium-ion battery (LIB) research, further development is essential to satisfy the growing demand for high energy and power densities, driven by applications such as high-end portable electronics and hybrid electric vehicles [1,2,3,4,5,6,7,8,9,10]. High-energy cathode [11,12,13,14] and anode materials [15,16,17,18] have been extensively explored and developed. In addition, innovative alloys [19,20] and metal oxides [21] also show significant potential as next-generation cathode materials. Graphite is currently the most common active anode material, mainly owing to its low average potential (0.1 V), structural stability, and cost-effectiveness; however, the low theoretical capacity of graphite anodes (approximately 372 mAh g^−1^) limits the overall energy density of LIBs. Conversely, silicon-based anodes provide an excellent theoretical capacity of approximately 4200 mAh g^−1^ [22,23,24] and are emerging as promising candidates for next-generation LIBs [25,26,27,28,29]. Despite their significant capacity potential, the commercialization of si-based anodes faces several important challenges. In particular, silicon-based anodes can undergo significant volume expansion of up to 300% during the lithiation process, which can cause significant mechanical stress within the anode material, resulting in particle failure, electrical contact loss, and ultimately, rapid capacity fading [30,31]. These mechanical failures not only degrade battery performance but also limit the overall lifespan of the battery. In addition, the repetitive volume fluctuations inherent in the silicon-based anode continue to destroy the solid–electrolyte interface (SEI) layer, which is critical for battery operation. During the initial charging phase, reduction reactions on the silicon-based anode surface induce the formation of an SEI, which serves as a protective interphase; however, this layer is continuously decomposed and modified by the action of mechanical stress during charging, resulting in consumption of the electrolyte and active lithium ions. This process therefore not only deteriorates the battery performance but also significantly shortens its cycle life [32]. Recent studies have sought to address these issues by improving SEI stability through the use of electrolyte additives and solid electrolytes, aiming to maximize the potential of silicon-based anodes in high-performance LIBs.

## 2. Si-Based Anode

**Figure 1:**
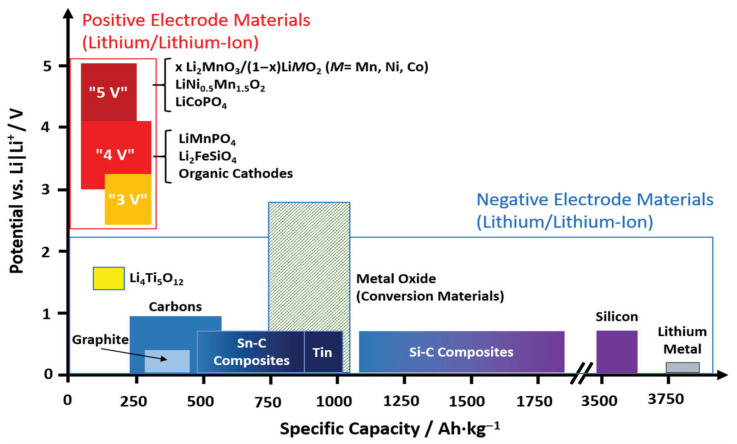
Voltage and capacity ranges of selected positive electrode and negative electrode materials currently considered for lithium-ion batteries (reprinted with permission from Ref. [34]; Copyright © 2022 Wiley).

The theoretical capacity of certain compounds used in silicon-based anodes is remarkably high. For example, that of Li_13_S_4_ is 3579 mAh g^−1^ (Figure 1) [7,33,34]. This capacity is almost 10 times higher than that of the commercial graphite anodes currently used in LIBs. The silicon-based anode has a lower potential difference (0.2–0.5 V) than a Li^+^/Li anode, resulting in a high energy density. This reduces the overall weight of the battery because a smaller quantity of active material is required during electrode fabrication. In addition, Si is the third most abundant element in the Earth’s crust, providing a significant economic advantage owing to lower material costs.

Despite these advantages, silicon-based anodes have limitations. In particular, the low electrical conductivity of silicon-based anodes (approximately 10^−3^ S/cm) inhibits efficient electron transport during charge and discharge cycles. Additionally, the Li diffusion coefficient in silicon is relatively low, estimated to be between 10^−14^ and 10^−13^ cm^2^/s. These factors limit the overall rate capabilities of silicon-based anodes in practical applications.

**Figure 2:**
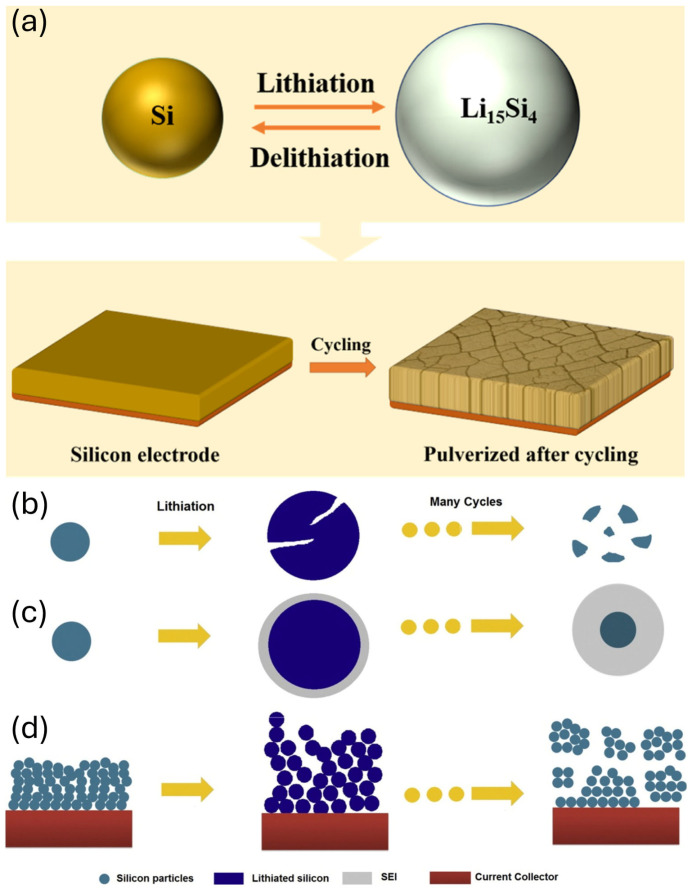
(**a**) Schematic of the silicon volume change reaction (reprinted with permission from Ref. [35]; Copyright © 2023 Elsevier). Silicon-based anode failure mechanisms. (**b**) Material pulverization. (**c**) Continuous SEI growth. (**d**) Morphology and volume expansion of the entire silicon-based anode (reprinted with permission from Ref. [33]; Copyright © 2012 Elsevier).

Additionally, the significant volume changes that occur during lithiation and delithiation cycles prevent silicon-based anodes from maintaining their capacity in the long term. These volume changes can reach 300% and cause significant mechanical stress within the anode material. In contrast to graphite, in which Li ions intercalate and deintercalate in a layered structure, Si undergoes an alloying reaction with lithium [35]. This alloying reaction can result in crack formation during repeated cycling, resulting in loss of electrical contact (Figure 2) [19,36,37,38]. Consequently, the cycle life of the silicon-based anodes is severely compromised, necessitating the development of strategies that enhance their stability and longevity.

Current research efforts aim to improve the cycling stability and overall performance of silicon-based anodes. Various methods have been explored, including the use of Si nanoparticles, the incorporation of electrolyte additives, the use of appropriate binders, and the development of active composite materials. The addition of small amounts of additives to the electrolytes has received particular attention. Several experimental studies have demonstrated that electrolyte additives can improve the electrochemical performance, thermal stability, and structural integrity of silicon-based anodes. Furthermore, these additives can also improve the formation and stability of the SEI layer, enhance ionic conductivity, and mitigate the adverse effects of volume expansion, thereby improving the overall performance and longevity of silicon-based anodes.

In summary, although silicon-based anodes offer significant advantages over their graphite counterparts, including higher capacity and energy density, inherent challenges remain to be overcome to ensure their successful commercialization in next-generation LIBs. Continued research on innovative materials and optimization strategies is expected to unlock the full potential of Si as a high-capacity anode material.

## 3. Electrolytes in Silicon-Based Anode

The mechanical stability of silicon-based anode systems during cycling is an important area of research. Numerous studies have modified the electrode configuration to improve their stability; however, repeated cracking and reconstruction of the SEI layer owing to the significant volume expansion associated with silicon-based anodes increases the thickness of the SEI layer [39], thereby increasing interfacial impedance and reducing battery capacity [40]. Therefore, controlling the electrolyte composition is essential for reducing SEI thickness and maintaining battery performance.

Upon cycling, the SEI layer on the silicon-based anodes evolved much more rapidly than that on the graphite anodes. Similarly to the SEI on graphite anodes, the decomposition of lithium ethylene dicarbonate (LEDC) produces Li_2_CO_3_ and other species; however, the decomposition reactions occur more readily on Si [41]. Decomposition of the LEDC exposed the silicon particle surface to the electrolyte, resulting in further electrolyte reduction. This results in the formation of a three-layered SEI on silicon composed of lithium silicate and silica near the silicon surface, an intermediate layer rich in Li_2_CO_3_ and LiF, and an outer layer primarily composed of LEDC [42,43,44].

**Figure 3:**
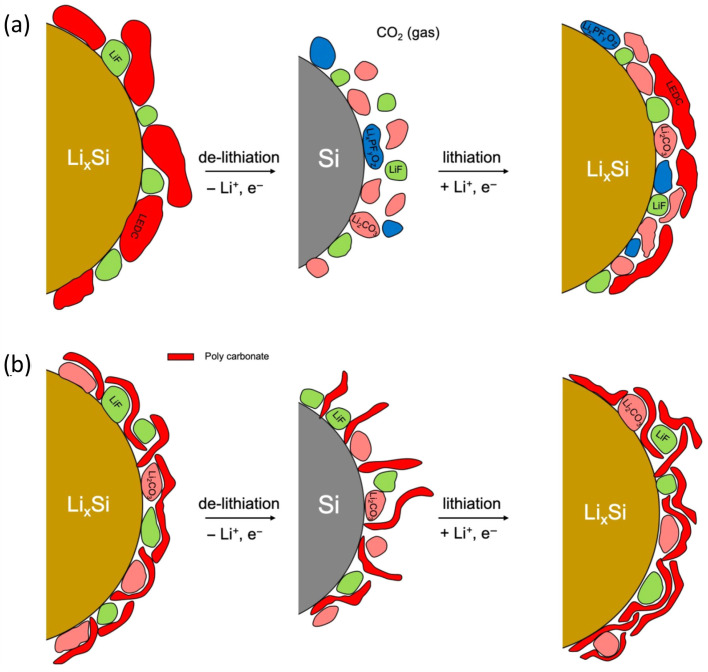
Schematic of the initial formation and growth of the SEI layers on silicon-based anodes in (**a**) EC-based electrolytes and (**b**) FEC-based electrolytes. The instability of the initially formed SEI component complicates the structure and composition of the SEI layer in cycling (reprinted with permission from Ref. [39]; Copyright © 2021 The Electrochemical Society).

The rapid decomposition of LEDCs at the silicon anode may arise from mechanical damage caused by large volume changes during the calcification and dissolution processes or catalytic decomposition by silica or lithium silicate. Mechanical damage caused by the expansion and contraction of the Si particles resulted in the formation of cracks in the rigid LEDC-based SEI layer, thereby increasing the surface area and thus the interaction with the electrolyte. This enhanced interaction accelerates the reaction between acid decomposition products (e.g., PF_5_, POF_3_, HF) of the LiPF_6_ and the LEDC, facilitating the rapid conversion of the LEDC and LiPF_6_ to LiF, CO_2_, fluorophosphoric acid, lithium alkoxide, and poly(ether) [45]. Alternatively, silica or lithium silicate may catalyze the thermal decomposition of LEDC, generating Li_2_CO_3_, Li_2_O, CO_2_, ethylene, and lithium carboxylates [46]. These reactions destabilize the SEI on silicon-based anodes relative to those on graphite, leading to the formation of thicker and more complex SEI layers over time (Figure 3).

This continuous decomposition increases the complexity of the SEI structure, which likely explains the variability in the SEI composition observed in several studies, owing to variations in the experimental conditions, such as the electrolyte concentration and cell composition. For example, laboratory studies typically employ a high electrolyte-to-electrode ratio, which can lead to higher impurity concentrations relative to the Si surface area, thereby accelerating SEI evolution [47,48].

To address these issues, considerable research efforts are being expended to develop advanced electrolytes that improve the stability and performance of silicon-based anodes. Electrolytes critically influence the long-term stability and performance of silicon-based anodes by determining the SEI chemistry. Innovative electrolyte designs include the integration of SEI-stabilizing additives, ionic liquids, solid electrolytes, and blending with high-concentration electrolytes. These strategies have the potential to alleviate the inherent problems associated with silicon-based anodes.

**Figure 4:**
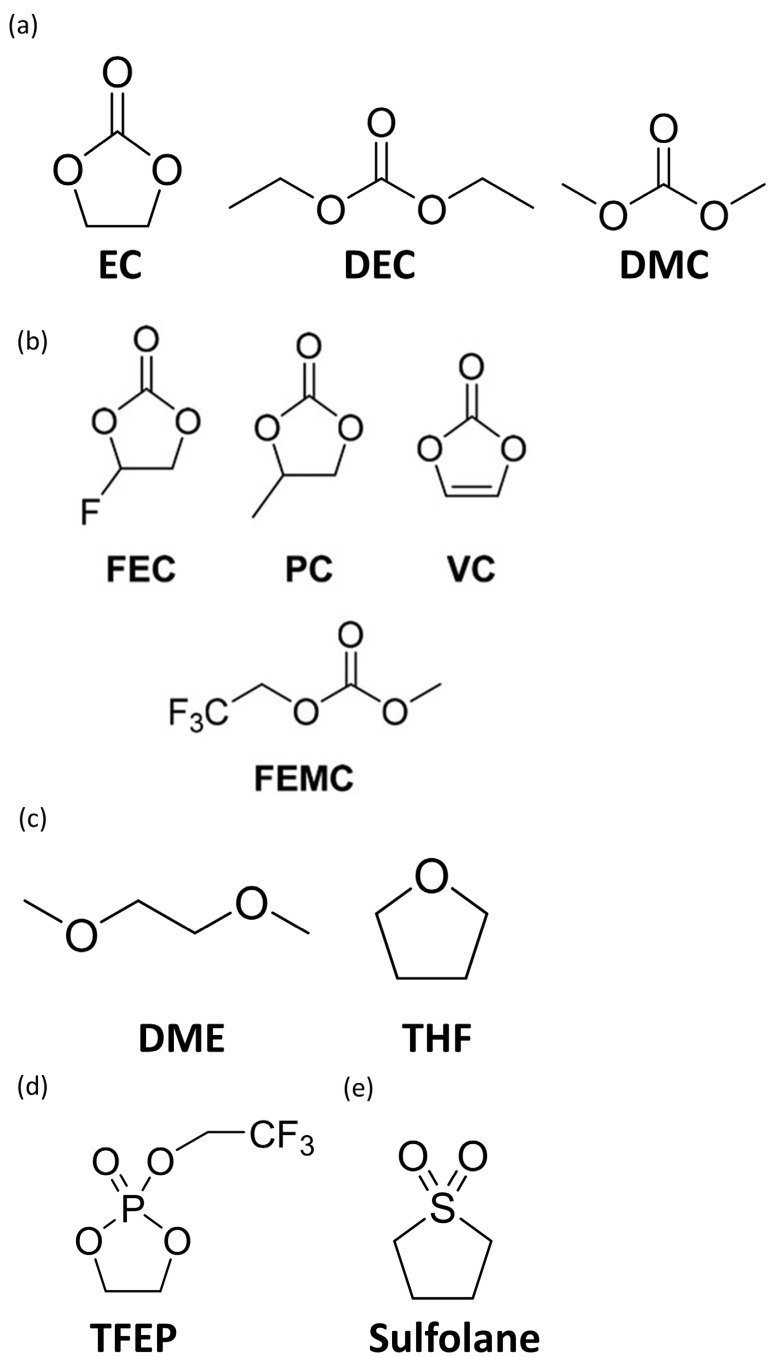
Chemical structure of selected (**a**,**b**) organic carbonate-based solvents. (reprinted with permission from Ref. [34]; Copyright © 2022 Wiley). (**c**) Ether-based solvents. (**d**) Phosphate-based solvents. (**e**) Sulfur-based solvents.

Solvents used in LIB electrolytes typically consist of a mixture of cyclic and linear carbonates. Cyclic carbonates such as ethylene carbonate (EC) have high dielectric constants and are stable under anodic currents; however, their high viscosities can hinder the diffusion of lithium ions when used alone, potentially reducing their ionic conductivities. To overcome this limitation, cyclic carbonates are often blended with linear carbonates such as diethyl carbonate (DEC) and dimethyl carbonate (DMC) to enhance the ionic mobility of the electrolyte (Figure 4a). Various solvents such as ether-, phosphate-, and sulfur-based compounds have also been explored as alternatives to carbon-based solvents (Figure 4). The type and composition of these solvents significantly influence the performance of the silicon-based anodes.

**Figure 5:**
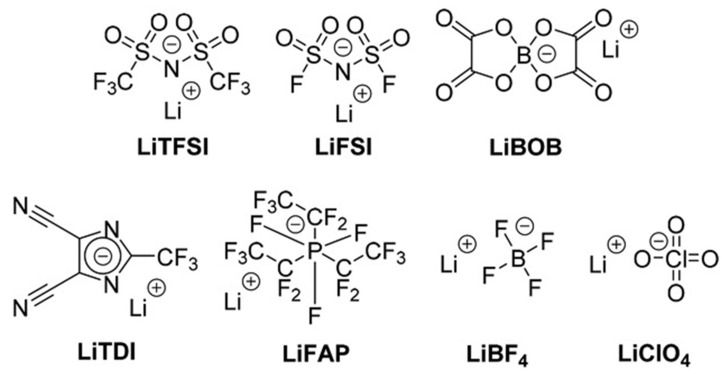
Chemical structures of selected lithium salts (reprinted with permission from Ref. [34]; Copyright © 2022 Wiley).

Lithium salts are crucial components of electrolytes in LIBs, facilitating the movement of lithium ions between the anode and cathode during charging and discharging cycles. Lithium hexafluorophosphate (LiPF_6_) is the most common lithium salt used in LIBs owing to its high ionic conductivity and stable chemical and electrochemical properties; however, LiPF_6_ reacts with moisture to form hydrofluoric acid (HF), which not only forms hydrogen gas and can lead to battery swelling but also decomposes the SEI layer, resulting in significant lithium depletion. Accordingly, alternative lithium salts, such as lithium bis(oxalato)borate (LiBOB), lithium bis(fluorosulfonyl)imide (LiFSI), and lithium thiophene-2,4-dicarboxylate (LiTDI) (Figure 5) [49,50,51,52], have attracted considerable research attention.

### 3.1. Additives

**Figure 6:**
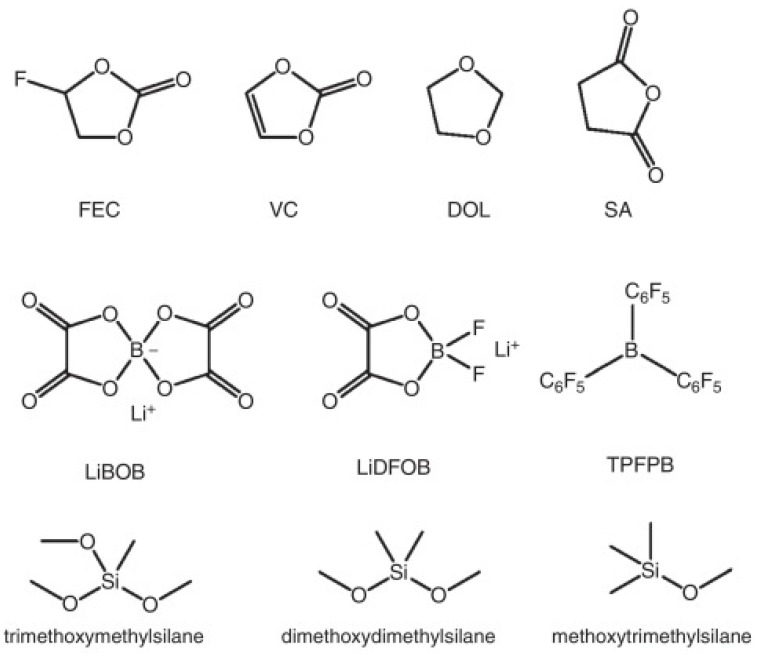
Chemical structures of electrolyte additives used with silicon-based anodes (reprinted with permission from Ref. [53]; Copyright © 2016 Elsevier).

Small quantities of liquid electrolyte additives are typically employed to preserve the overall characteristics of the electrolyte while introducing specific enhancements at a relatively low cost. These additives allow the performance of silicon-based anodes to be optimized by addressing inherent challenges associated with Si lithiation while maintaining the desired properties of the electrolyte (Figure 6) [53].

The properties of the SEI layer depend primarily on the composition of the electrolyte, particularly the additives. These additives not only form useful compounds that modify the SEI layer through reductive decomposition but also influence specific electrochemical behaviors at the electrode interfaces. The solvated structures of the interfaces significantly impact the battery performance, helping to optimize the SEI and improve the overall efficiency.

These additives are designed to decompose before the electrolyte during the initial formation of the SEI layer. This pre-decomposition generated reactive species that contributed to the formation of a stable and protective SEI layer. Thus, additives exert various effects that significantly improve battery performance.

Additives can enhance the electrochemical stability window of the electrolyte, thus enabling the battery to operate effectively at higher voltages while avoiding detrimental side reactions. Other additives are designed to improve the thermal stability of the electrode, mitigating the risks associated with elevated temperatures that can cause battery failure or degradation.

The incorporation of flame-retardant additives to reduce the flammability and thermal runaway potential and thus enhance the overall safety profile of LIBs is a critical safety consideration, particularly in high-energy applications. This safety enhancement is particularly important for silicon-based anodes, which are prone to mechanical failure owing to volume expansion during cycling.

Electrolyte additives can also alleviate the volume expansion of silicon-based anodes during the charging and discharging processes. These additives modify the electrochemical environment within the electrolyte, thereby stabilizing the SEI layer and minimizing the mechanical stress on the silicon-based anode, which improves the cycle life and overall performance.

In summary, the strategic incorporation of liquid electrolyte additives is a promising approach for enhancing the performance and safety of silicon-based anodes in LIBs. Continued exploration of novel additives and their mechanisms of action has considerable potential to achieve advancements that further improve battery technologies. The roles and electrochemical performance of each additive are summarized in Table 1.

#### 3.1.1. VC

**Figure 7:**
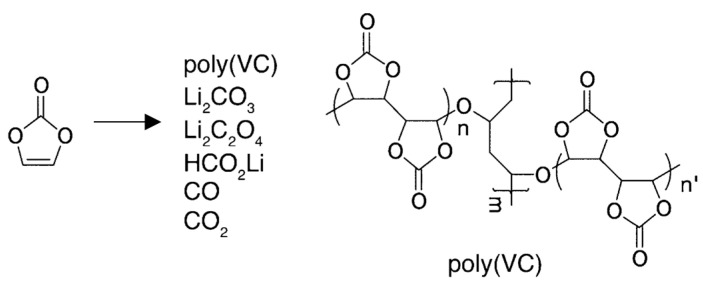
Proposed VC reduction products. A possible structure for a cross-linking site of poly(VC) is shown (reprinted with permission from Ref. [64]; Copyright © 2016 American Chemical Society).

Vinylene carbonate (VC) is a well-understood electrolyte additive that offers several advantages in silicon-based anodes. VC contains a double bond that decomposes to form various reactive intermediates, including lithium carbonate (Li_2_CO_3_) and poly(vinylene carbonate) (poly(VC)) (Figure 7) [64]. These products facilitate the formation of a stable SEI layer, which is crucial for maintaining the battery performance.

Chen et al. [54] demonstrated that the incorporation of 1 wt.% VC into a 1 M LiPF_6_ electrolyte solution composed of a 1:1 *v*/*v* mixture of EC and DMC increased the initial Coulombic efficiency (ICE) of the solution from 67.9% to 72.5%. Notably, the reversible capacity of the silicon-based anode in the electrolyte with added VC remained stable at approximately 2000 mAh g^−1^ for up to 200 cycles, gradually decreasing to over 500 mAh g^−1^ after 500 cycles. Furthermore, Leveau et al. [65] and Dalavi et al. [56] improved the electrochemical performances of silicon nanowires and nanosilicon films, respectively, using VC as an additive.

Despite its benefits, VC has drawbacks, including its increased impedance and significant internal resistance. Jaumann et al. [66] found that while VC afforded superior lifespan and efficiency compared to other additives, such as fluoroethyl carbonate (FEC), the resulting SEI layer exhibited structural defects that resulted in high resistance to Li^+^ migration. This limitation renders VC less suitable for high-power applications, where rapid ion transport is essential.

#### 3.1.2. Fluoroethylene Carbonate

**Figure 8:**
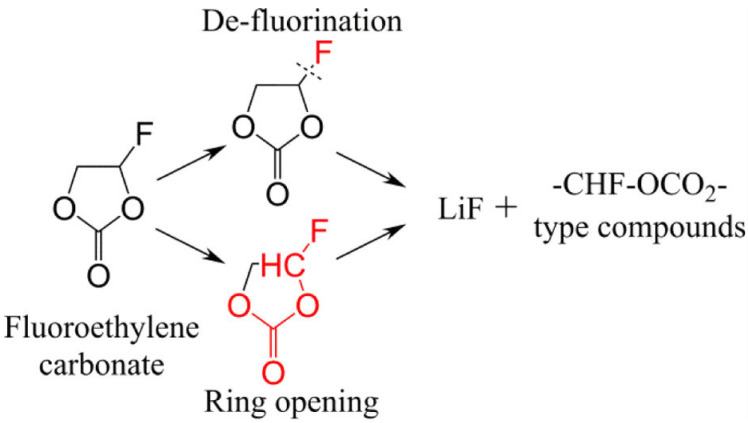
Possible FEC decomposition reactions and products (reprinted with permission from Ref. [71]; Copyright © 2015 Chemistry of Materials).

FEC is another notable additive that has garnered attention because of its role in enhancing the performance of silicon-based anodes. The SEI layer formed in the presence of FEC was characterized by a different set of mechanical properties than that formed in the presence of VC. FEC undergoes defluorination at approximately 0.9 V (vs. Li^+^/Li), generating LiF (Figure 8) [49,67,68,69], which is a critical component of the SEI layer. Owing to its high mechanical strength and excellent insulating characteristics, LiF suppresses suppressing electrode degradation during the significant volume changes that occur during charge and discharge cycles [70,71,72].

Kim et al. [57] found that incorporating 3 wt.% FEC into a 1.3 M LiPF_6_/EC-DEC electrolyte formulation afforded a less porous SEI layer on a silicon thin-film electrode. This SEI layer was rich in stable compounds such as LiF, which enhanced capacity retention during cycling. Although the SEI formed using FEC was less flexible than that formed using VC, it demonstrated superior ionic conductivity, which is critical in high-power applications [56].

Domi et al. [58] demonstrated that combining VC and FEC additives effectively improved the electrochemical performance of silicon-based anodes, even in the absence of conventional binders and conductive additives [59]. This synergistic effect demonstrates the potential of tailoring electrolyte formulations to optimize the performance of silicon-based anodes in LIBs.

Despite these advantages, FEC can lead to the formation of a thicker SEI layer with correspondingly higher internal resistance, while its relatively low thermal stability can limit its performance at high temperatures. Additionally, FEC consumes a significant amount of lithium during SEI formation, thereby reducing the ICE.

Therefore, although FEC improves the performance of silicon-based anodes, further research is needed to overcome its limitations and optimize its use in broader applications.

#### 3.1.3. Silane Additives

The reduction reactions of lithium ions with silicon oxide (-Si-O-Si-) and silanol (-Si-OH) groups on the surfaces of silicon-based anodes can result in irreversible capacity loss through reduction reactions with lithium ions. Surface modification with alkoxysilane additives has emerged as a promising strategy to address this issue and enhance the electrochemical performance of silicon-based anodes.

**Figure 9:**
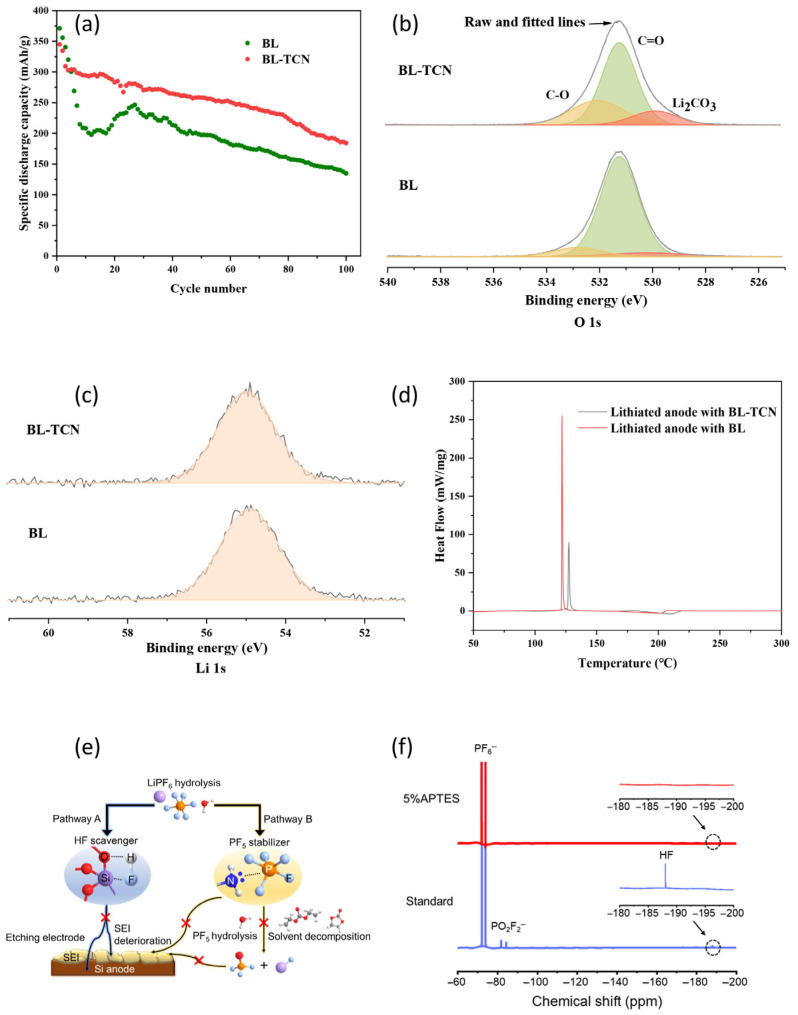
(**a**) Cycle performance of the cell with various electrolyte formulations. (**b**) O 1 s XPS spectra of the anode after 15 cycles with BL and BL-TCN electrolytes (BL: blank electrolyte). (**c**) Li 1 s XPS spectra of the anode after 15 cycles with BL and BL-TCN electrolytes. (**d**) DSC curve of lithiated anode mixture using various electrolytes at 6 °C/min (BL: blank electrolyte) (reprinted with permission from Ref. [61]; Copyright © 2022 MDPI). (**e**) Schematic of the HF removal and PF_5_ stabilization mechanism using the APTES additive. (**f**) F NMR spectra of the electrolyte with and without 5 wt.% APTES with 300 ppm deionized water (reprinted with permission from Ref. [62]; Copyright © 2022 American Chemical Society).

Zhang et al. [61] incorporated a silane-based polymer, (2-cyanoethyl) triethoxysilane (TCN), into a commercial electrolyte at a 5% volume ratio. TCN resulted in the formation of a more stable SEI layer during long-term charge–discharge cycles, significantly enhancing the cycling stability (Figure 9a). X-ray photoelectron spectroscopy (XPS) revealed a high concentration of Li_2_CO_3_ in the SEI film formed in the TCN-containing electrolyte (Figure 9b,c), which improved the mechanical stability of the SEI layer. Furthermore, differential scanning calorimetry (DSC) indicated that the addition of TCN substantially inhibited the exothermic reactions between the lithiated anode and the electrolyte, thereby reducing the heat release (ΔH decreased from 250.48 to 151.71 J/g) and increasing the reaction initiation temperature (T_o_ increased from 122.22 to 127.07 °C), indicating an improvement in thermal stability. Thermogravimetric analysis (TGA) further confirmed these findings, showing a slower rate of mass loss in the TCN-containing samples, which was consistent with DSC (Figure 9d). Furthermore, the addition of TCN increased the activation energy (Ea) of the lithiated anode–electrolyte mixture from 68.46 to 91.32 kJ/mol, which suggests that TCN additives enhanced the safety of silicon-based anode LIBs. Tian et al. [62] (Figure 9e) expanded the investigation of organosilicon-containing electrolytes using 3-aminopropyltriethoxysilane (APTES) as an additive. The addition of 5 wt.% APTES to the electrolyte eliminated the exothermic peaks typically observed with carbonate-based electrolytes and significantly improved the thermal stability of the lithiated silicon-based anode (Figure 9f). This enhanced thermal stability was attributed to the reduction of PF_5_/HF by APTES in the electrolyte, thereby minimizing the detrimental effects of LiPF_6_ hydrolysis and solvent decomposition. Additionally, APTES formed a protective polymer layer on the silicon surface that prevented direct contact between the lithiated silicon and the electrolyte while promoting the formation of thermally stable SiO_2_, which provided a physical barrier against further electrolyte degradation. These results indicate that silane-based additives contribute to both the electrochemical and thermal stability of silicon-based anodes through the formation of durable SEI layers, thereby mitigating their mechanical and thermal limitations. Such Si anodes effectively address the mechanical and thermal challenges associated with silicon-based anodes.

In addition, research on other alkoxysilane additives has shown promise. For instance, trimethoxymethylsilane (TMMS) was investigated in nanocrystalline silicon thin-film electrodes in a 1 M LiPF electrolyte solution composed of EC and DEC. Attenuated total reflectance Fourier transform infrared spectroscopy demonstrated that TMMS additives promote stable cycling performance between 0 and 1.5 V vs. Li/Li^+^, achieving a reversible capacity of 2400 mAh g^−1^ over 200 cycles. This improvement is attributed to the formation of a stable SEI layer rich in organic compounds such as alkyl carbonates and carboxylic acid metal salts, along with phosphorus- and fluorine-containing species, which extend the cycle life and improve stability [53].

Although the low dielectric constants of siloxanes and silanes can limit their Li ion transport abilities, they also foster favorable electrode/electrolyte interfaces in silicon-based anodes [22]. In particular, alkoxy silane-based additives chemically interact with hydroxyl groups on the silicon-based anode surface, thereby suppressing mass accumulation in the silicon film electrodes and enhancing the cycle life [73]. The formation of organosilicon compounds also plays a crucial role in preventing severe cracking of Si films and reducing the interfacial resistance [74].

Additional studies of silane additives with multiple alkoxy substitutions have evaluated the impact of various substituents on performance [73]. The alkoxysilane group is effective for constructing stable SEI layers in LIBs equipped with silicon anodes, demonstrating the potential of these additives to advance the commercial viability of silicon-based battery technologies [53]. In addition to silane-based compounds, various alternative additives have been developed to further enhance the SEI functionality and thermal stability.

#### 3.1.4. Other Additives

In recent studies, various additives beyond the aforementioned compounds have garnered attention for their potential to enhance the performance of silicon-based anodes. One such additive, dimethylacetamide (DMAA), has excellent solubility in both hydrophilic and hydrophobic solvents. DMAA plays a critical role in the lithium alloying process with silicon, forming a complex with PF_5_ generated through the dissociation of LiPF_6_ and thereby suppressing electrolyte decomposition and mitigating SEI layer degradation. Zhu et al. [60] demonstrated the application of DMAA as an electrolyte additive for use with nano-silicon electrodes in half-cell configurations. DMAA effectively inhibited electrolyte decomposition by facilitating the formation of a stable SEI layer on the surface of the silicon-based anodes at elevated potentials. Furthermore, the incorporation of DMAA into electrolytes containing FEC enhances the electrochemical properties of silicon-based anodes. For instance, in a 1 M LiPF_6_ electrolyte mixed with EC, DMC, DEC, and FEC in a 3:3:1 volume ratio, the silicon-based anodes exhibited an ICE of 80.16% and a capacity retention of over 80% after 500 cycles.

The development of electrolytes that rely on a single additive to achieve satisfactory overall electrochemical performance presents significant challenges. The broader context of LIB energy density must be considered rather than focusing solely on silicon-based anodes. For example, when designing electrolytes for entire cells that use Ni-rich cathodes along with silicon-based anodes, the cathode electrolyte interphase (CEI) must be stabilized while ensuring the stability of the SEI layer on Si. Consequently, optimizing overall battery performance may require a tailored approach involving a blend of multiple additives.

The use of auxiliary additives to address the low thermal stability of FEC in silicon-based batteries has been explored. Jo et al. [75] investigated a binary additive system comprising FEC and di(2,2,2-trifluoroethyl) carbonate (DFDEC) at concentrations of 10 and 1 wt.% within an EC/EMC electrolyte in a half-cell configuration with a Si/graphite composite electrode. The binary additive system afforded a significant enhancement in the electrode capacity during cycling relative to that achieved with configurations employing FEC alone.

Additionally, Han et al. [76] used a dual-function additive, lithium fluoromalonato-(difluoro)borate (LiFMDFB), to stabilize both Li-rich cathodes and Si-graphite anodes in full-cell applications. LiFMDFB facilitates the formation of a stable CEI that protects the lithium-rich cathode while also tuning the SEI characteristics at the Si/graphite interface. With the incorporation of LiFMDFB, the Si/graphite particles maintained a uniform SEI even after 200 cycles, which was attributed to synergistic interactions between the FMDFB anions and FEC. The full cell achieved an impressive capacity retention of 85% after 100 cycles, along with a Coulombic efficiency of 99.5% and an energy density of 400 Wh/kg.

**Figure 10:**
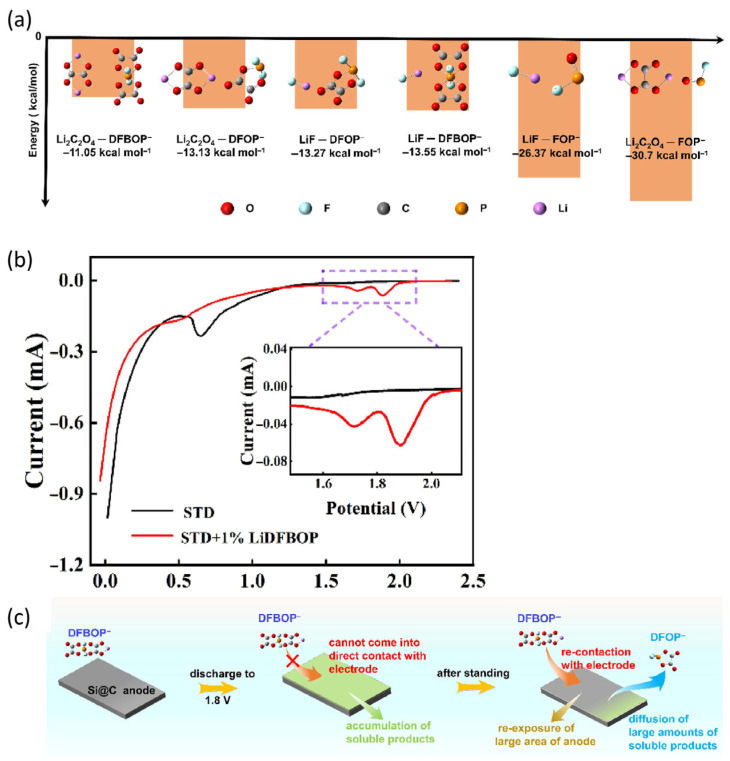
(**a**) Linear sweep voltammetry curves of Li//Si@C half-cells with standard electrolyte (STD) and STD + 1% LiDFBOP electrolytes. (**b**) Binding energies of decomposition products with various solvation structures. (**c**) Schematic of effects of cut-off voltage control to suppress full decomposition of LiDFBOP in intermittent discharge mode (reprinted with permission from Ref. [63]; Copyright © 2024 Elsevier).

Chunglei et al. [63] explored the use of lithium difluorobisoxalate phosphate (LiDFBOP) as a novel electrolyte additive in silicon–carbon (Si@C) anodes. LiDFBOP enhanced SEI film formation owing to its preferential reductive decomposition, which generates inorganic compounds such as LiF and Li_2_C_2_O_4_ (Figure 10b). These compounds increase the ionic conductivity and strengthen the mechanical properties of the SEI layer, thereby reducing the volume expansion of Si during the lithiation and delithiation processes. Intermittent discharge in specific voltage ranges optimized the decomposition efficiency of LiDFBOP and improved SEI formation. The application of intermittent discharge at 1.8 V (Figure 10a,c) resulted in the continuous decomposition of LiDFBOP, leading to more uniform Li^+^ intercalation and enhanced electrode stability. The optimized SEI film afforded superior control over internal stresses and minimized Si particle pulverization, thereby improving the electrochemical performance of the Si-C anode. This strategy enabled more efficient utilization of LiDFBOP, reducing both cost and material waste while significantly improving the cycle life and capacity retention. Optimizing the decomposition conditions of additives such as LiDFBOP may therefore advance the development of high-performance silicon-based batteries and may be particularly effective in mitigating the challenges posed by volume expansion.

## 4. New Challenges in Electrolytes: Solid-State Electrolytes

In addition to electrolyte additives, solid electrolytes (SEs) are being actively developed to improve the performance of silicon-based anodes. Many studies have investigated lithium-metal anodes and anode-free configurations; however, recent studies demonstrated the potential of applying SEs to silicon anodes. The transition from organic liquid electrolytes to SEs offers important advantages that ameliorate the inherent fire risk. SEs inherently possess higher energy density and reduce flammability, thereby eliminating the need for additional safety features. Consequently, SEs have attracted considerable attention as viable alternatives for next-generation batteries.

**Figure 11:**
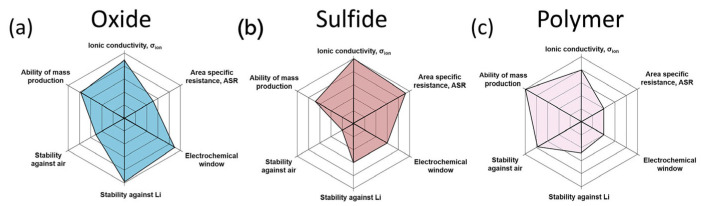
(**a**–**c**) Properties of representative solid-state electrolytes (reprinted with permission from Ref. [77]; Copyright © 2019 Elsevier).

SEs can be classified as oxide-based, sulfide-based, or polymer-based electrolytes (Figure 11a–c) (Table 2) [77].

### 4.1. Oxide-Based Solid Electrolytes

**Figure 12:**
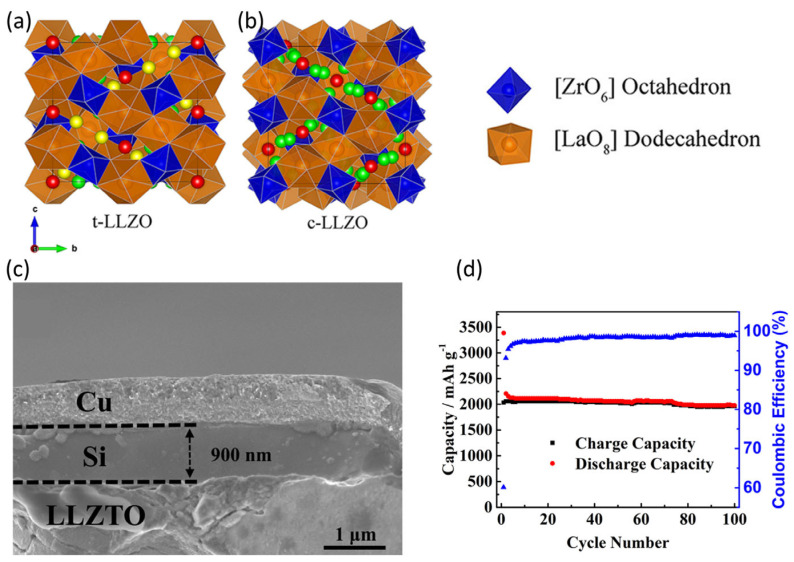
The spatial structures of the (**a**) tetragonal and (**b**) cubic phases of LLZO (reprinted with permission from Ref. [82]; Copyright © 2018 American Chemical Society). (**c**) The structure of an all-solid-state battery assembled with a silicon-based anode and LLZTO electrolyte; (**d**) electrochemical cycling lithium storage performance of battery with 45 nm-thick Si layer (reprinted with permission from Ref. [79]; Copyright © 2018 American Chemical Society).

Oxide-based SEs are suitable for silicon-based LIBs owing to their wide electrochemical stability window, excellent ionic conductivity, and high mechanical strength. Murugan et al. [78] showed that garnet-structured lithium lanthanum zirconates (LLZO) exhibit excellent ionic conductivity of 3 × 10^−4^ S cm^−1^. Garnet-based electrolytes characterized by rectangular or cubic crystal structures currently dominate the field of silicon-based LIBs (Figure 12a,b) [82]. In particular, the electrolytes with cubic phases show improved adaptability to lithium-ion migration, providing higher ionic conductivity [83,84]. Chen et al. [79] doped LLZO with Ta to produce LLZTO oxide SEs, producing an all-solid battery assembled with a silicon-based anode that exhibited excellent electrochemical performance (Figure 12c,d). Tantalum doping significantly enhances the ionic conductivity of LLZO, which in turn improves the performance of silicon-based anodes. The increased ionic conductivity increased the rate of lithium-ion transport, thereby improving both the cycling performance and stability of the anode. Moreover, the enhanced lithium-ion mobility at the interface mitigates issues related to the volume expansion of silicon during cycling and reduces SEI formation, resulting in improved structural integrity and longer battery life. All-solid-state batteries that combine LLZTO electrolytes with silicon-based anodes exhibit excellent electrochemical performance, high energy density, and stable cycling. This underscores the importance of oxide-based SEs, particularly tantalum-doped electrolytes, in the development of next-generation batteries with silicon-based anodes.

### 4.2. Sulfide-Based Solid Electrolytes

The ionic conductivity and mechanical ductility of sulfide-based SEs are superior to those of other SEs and can accommodate the significant volume changes associated with silicon-based anodes. This study explores the use of lithium sulfide (Li_2_S) as a cathode material in all-solid-state batteries. The use of silicon-based anodes avoids interface problems commonly encountered in lithium metal anodes; however, significant problems arise at the interface between silicon-based anodes and SEs owing to unwanted side reactions caused by chemical and electrochemical instabilities. Mitigating these interfacial problems is essential to optimize battery performance. For example, Xiaoyan et al. [85] used Li_2_S cathodes to achieve high energy density while mitigating interfacial problems. A combination of lithium sulfide cathodes, Li_7_P_3_S_11_ (LPS) electrolytes, and silicon-based anodes afforded a more stable interface and improved electrochemical performance.

**Figure 13:**
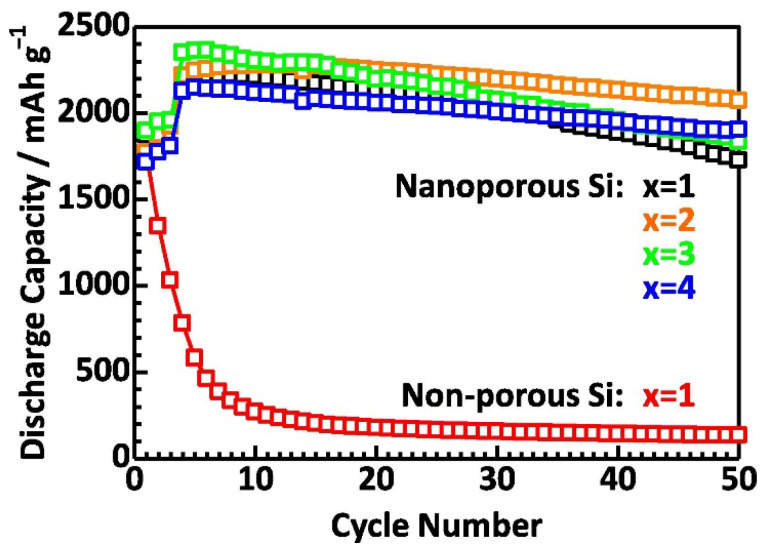
Comparison of the cycling performance of half-cells employing nanoporous silicon with varying contents of CA (composite ratio of Si:SE:CA = 4:6:x *w*/*w*). Data from a half-cell using nonporous silicon at x = 1 are also included for reference (reprinted with permission from Ref. [87]; Copyright © 2022 Elsevier).

In addition, Xiaoyan et al. coated Si nanoparticles with LPS in situ, thereby enhancing the interfacial contact, alleviating Si volume expansion, and improving lithium-ion diffusion during cycling. These coated nanoparticles achieved an initial discharge capacity of 316.9 mAh g^−1^ in an all-solid Li_2_S/Si full cell, stabilized to 179.4 mAh g^−1^ after 20 cycles, demonstrating that interface stability is crucial in maintaining the performance of the all-solid-state battery. Restricting SEI formation within a two-dimensional plane, effectively limiting side reactions, is another promising strategy for improving interface stability. Meng et al. [80] investigated the interfacial problem with an anode composed of 99.9% wt.% micron-sized Si using lithium thiophosphate (Li_6_PS_5_Cl) as an electrolyte, demonstrating that the initially low ICE improved over the subsequent cycle, providing a stable interface that minimized side reactions throughout the cycle. With the exception of conductive additives such as carbon, electrolyte decomposition decreased during the initial cycle, further improving the ICE. Carbon-based materials are often introduced into composite electrodes together with sulfide electrolytes to further improve the conductivity of silicon-based anodes. Nanosilicon has low electrical conductivity; thus, the introduction of carbon improves conductivity and facilitates stress diffusion arising from Si volume expansion. However, the presence of carbon can accelerate the decomposition of solid electrolytes in silicon-based anodes. Okuno et al. [86] developed a composite electrode consisting of nanosilicon, Li_3_PS_4_ electrolyte, and acetylene black (AB), wherein an increase in the AB content formed additional electron paths and prevented Si particle aggregation. This configuration, with a Si:Li_3_PS_4_:AB weight ratio of 4:6:2, exhibited a discharge capacity of 2071 mAh g^−1^ and maintained 91% of the capacity over 50 cycles, demonstrating its superlative electrochemical stability (Figure 13). Despite these advantages, sulfide-based solid electrolytes are highly vulnerable to hydrolysis reactions with moisture and oxygen that release hydrogen sulfide (H_2_S), which poses environmental and safety risks. Strictly inert atmospheric conditions are therefore required to prevent decomposition during the preparation and handling of these materials.

### 4.3. Polymer-Based Solid Electrolytes

**Figure 14:**
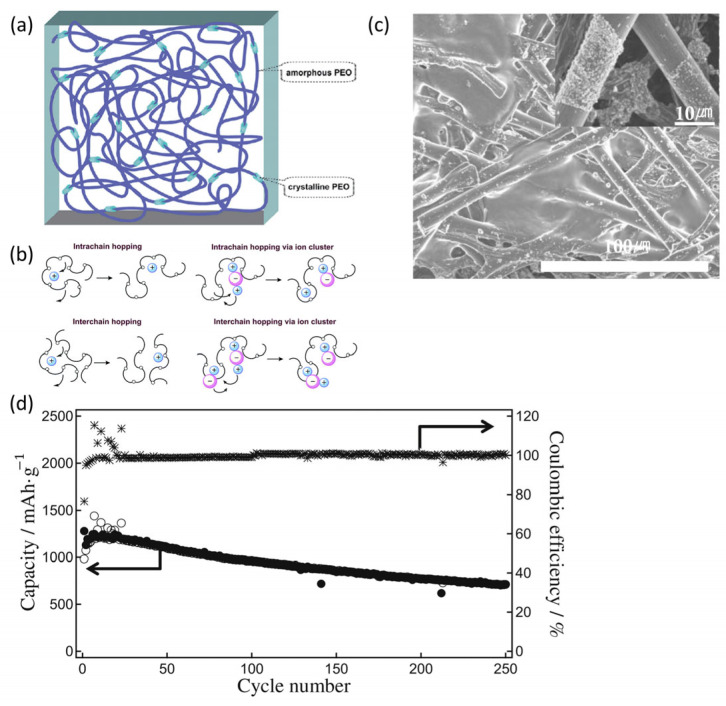
(**a**) The structure and (**b**) internal ion transport mechanism of PEO (reprinted with permission from Ref. [35]; Copyright © 2023 Elsevier). (**c**) SEM image of the silicon–carbon composite anode surface paired with the PEO electrolyte membrane, and (**d**) cycling performance of the Si/C/CP (CP: carbon paper) electrode using PEO_18_LiTFSI electrolyte at 0.1 A g^−1^ and 60 °C, evaluated within a voltage range of 0.02–1.5 V. ●: lithium insertion into Si/C/CP, ○: lithium stripping from Si/C/CP (reprinted with permission from Ref. [81]; Copyright © 2014 Elsevier).

Polymer-based solid electrolytes offer low mass, excellent interface contact, flexibility, and superior processability. The most common polymers used in electrolyte membrane matrices that are compatible with silicon-based anodes include polyethylene oxide (PEO) and polyvinylidene fluoride [87,88]. Above the glass-transition temperature, PEO exists in an amorphous state that facilitates lithium-ion conduction; however, at room temperature, it adopts a crystalline form that restricts ion mobility, thereby reducing the ionic conductivity (Figure 14a). The ethylene oxide repeating units in PEO have high donor numbers and flexibility, facilitating lithium-ion transport within the polymer matrix (Figure 14b). Si et al. [81] explored the application of silicon-based anodes in solid-state batteries with PEO-based electrolytes and demonstrated stable performance with a PEO film containing a single additive, lithium bis(trifluoromethanesulfonyl)imide (LiTFSI), achieving an ICE of 77%. At 60 °C and a current density of 100 mA g^−1^, a reversible capacity of 710 mAh g^−1^ was maintained after 250 cycles (Figure 14c,d).

The incorporation of carbon fibers into a carbon paper substrate provided optimal contact with the Si-active material, effectively mitigating the volume expansion of the electrode and ensuring structural stability throughout the cycling. Similarly, all-solid-state batteries using silicon-based anodes paired with lithium iron phosphate (LiFePO_4_) cathodes yielded promising electrochemical outcomes, with LiFePO_4_ retaining a specific capacity of 150 mAh g^−1^ after 50 cycles, comparable to that of traditional liquid cell configurations. Si et al. further demonstrated that the modifications of silicon-based anode structures designed to alleviate volume expansion and enhance electrochemical stability in liquid LIBs are equally applicable to PEO-based polymer electrolyte systems [35]. Although polymer electrolytes incur low production costs and exhibit ease of preparation and high flexibility, their inherently low room-temperature ionic conductivities necessitate their combination with additional additives to optimize their performance.

### 4.4. Interfacial Challenges Between Solid Electrolytes and Silicon Anodes

The interface between SEs and silicon-based anodes presents several critical challenges, including severe volume expansion of Si, unstable SEI formation, undesirable side reactions, and limited lithium-ion transport [89]. To address these issues, three principal strategies have been proposed:

1. Electrolyte Optimization:
  Quasi-solid or composite electrolytes incorporating dual lithium salts, flexible polymer matrices, or ceramic additives such as LLZTO [90] and propylene carbonate (PC) [91] have been developed to form mechanically robust and ionically conductive interfaces. These systems alleviate SEI instability, accommodate Si volume changes, and improve capacity retention and ICE [92].
2. Artificial SEI Layers:
  Artificial interlayers—including in situ-formed LiF-rich SEI films [93], LiAlO_2_ coatings [94], and LLZTO-modified surfaces [95]—have demonstrated effectiveness in suppressing electronic leakage, mitigating interfacial side reactions, and enhancing cycling stability. Both in situ and ex situ techniques have been employed to improve the structural integrity of the Si/SE interface.
3. Anode Structural Design:
  Nanoengineering approaches, such as the development of thin-film Si [96], columnar Si structure [97], Si@C composites [92], metal–organic framework (MOF)-derived Si@MOF architectures [98], and vertical graphene–Si hybrids [99], have been shown to effectively buffer volume changes, preserve interfacial contact, reduce interfacial resistance, and improve mechanical compliance with the electrolyte.

Collectively, these strategies contribute to enhanced interfacial stability, reduced resistance, and prolonged electrochemical performance in silicon-based all-solid-state batteries.

## 5. Conclusions

Advances in LIB technology have significantly expanded the potential applications of silicon-based anodes. Although Si is an attractive anode material owing to its excellent lithium storage capacity, its application is hindered by several challenges arising from the unique mechanisms operating in silicon. In particular, the significant volume expansion and pulverization during the lithiation and delithiation processes, instability of the SEI, and morphological changes at the electrode level hinder the development of anodes with high Si contents, thereby limiting their commercial viability. Numerous studies have sought to overcome these obstacles to satisfy the unique requirements of silicon-based materials by employing various strategies, including controlling the microstructure of the active materials and developing composites based on carbon and metals. In particular, the optimization of the electrolyte composition has drawn attention as a means of improving the performance and stability of silicon-based anodes.

This study examined the potential of silicon-based anodes, reviewing various studies aiming to facilitate the commercialization of these materials, with a particular focus on electrolyte additives and next-generation all-solid-state batteries. Further research is essential for realizing the full potential of silicon-based LIBs. Optimizing the structure of silicon-based anodes and developing electrolyte systems compatible with silicon-based anodes should be major areas of focus. The formation of a high-quality SEI layer on the electrode surface is essential for improving the performance and cycle life of silicon-based anodes. Combining several electrolyte additives, such as dual or complex formulations, will likely prove an effective strategy to achieve desirable SEI properties.

Considerable challenges remain in the optimization of silicon-based anodes; however, innovations in cathode materials and enhancements to current collector structures present significant opportunities. These integrated advances can facilitate the widespread adoption of silicon-based anodes in next-generation batteries, which can ultimately improve the performance of battery-powered electric vehicles and electronic devices. As research continues to address the challenges related to silicon-based anodes, the prospects for their application in high-performance batteries remain optimistic.

While considerable challenges persist in optimizing silicon-based anodes, concurrent innovations in cathode materials and current collector architectures offer promising avenues to overcome these limitations. The integration of these advancements is expected to accelerate the practical implementation of silicon-based anodes in next-generation lithium-ion batteries, thereby enhancing the overall performance of electric vehicles and portable electronic devices. With continued research efforts, the outlook for the widespread application of silicon-based anodes in high-performance energy storage systems remains highly encouraging.

## Figures and Tables

**Table 1:** Comparison of various additives (all data are based on individual references). Estimated cost values are approximate and reflect research-grade prices from chemical suppliers and public listings as of 2023–2024. They are intended for relative comparison only.

Additive	SEI Stability	ICE (%)	Cycling Performance	Estimated Cost	References
VC	Impermeable SEI with low impedance growth during cycling	67.9 → 72.5	~2000 mAh g^−1^ (200 cycles), >500 mAh g^−1^ (500 cycles)	Low	[54,55]
FEC	Forms LiF-rich SEI; mechanically robust	88.7	Maintains > 90% capacity; >99% efficiency over extended cycles at high rate	Low	[56,57,58,59]
DMAA	Forms dense, stable SEI and suppresses side reactions	~80.2	>80% retention after 500 cycles	Low	[60]
TCN	table SEI rich in Li_2_CO_3_	-	Reduced capacity fade and improved stability	Midium	[61]
APTES	Forms protective layer with SiO_2_-rich SEI	-	Enhances cycling stability with 5 wt% addition	Midium	[62]
LiDFBOP	Forms LiF/Li_2_C_2_O_4_ SEI; reduces volume expansion	-	Improves Li^+^ diffusion and preserves structural integrity	High	[63]

**Table 2:** Comparison of three types of SEs (all data are compiled from the cited references). Estimated cost values are approximate and correspond to research-grade prices available from chemical suppliers and public listings as of 2023–2024. The comparisons are intended to provide general guidance on the practical characteristics of each SE type.

SE Type	Ionic Conductivity (S/cm)	Mechanical Stability	Interfacial Compatibility with Si	Estimated Cost	References
Oxide (LLZO)	~3 × 10^−4^	High (brittle)	Moderate (requires doping/coating)	Medium	[78,79]
Sulfide (Li_6_PS_5_Cl)	~10^−3^~10^−2^	Moderate (ductile, sensitive)	Good (enhanced with coatings)	High	[80]
Polymer (PEO)	~10^−6^ at RT; better at 60 °C	Low (soft, flexible)	Excellent (good interface contact)	Low	[81]
